# Long Noncoding RNA EMX2-AS Facilitates Osteoblast Differentiation and Bone Formation by Inhibiting EMX2 Protein Translation and Activating Wnt/*β*-Catenin Pathway

**DOI:** 10.1155/sci/4397807

**Published:** 2024-11-26

**Authors:** Linyuan Fan, Li Zhang, Xin Zhang, Wei Wei, Zhaohui Liu

**Affiliations:** Department of Gynecology, Beijing Obstetrics and Gynecology Hospital, Capital Medical University, Maternal and Child Health Care Hospital Beijing, Beijing 100026, China

**Keywords:** EMX2, lncRNA EMX2-AS, long noncoding RNAs, mesenchymal stem cells, osteoblast differentiation, Wnt/*β*-catenin

## Abstract

Long noncoding RNAs (lncRNAs), as a potentially new and crucial element of biological regulation, have gained widespread attention in recent years. Our previous work identified lncRNA empty spiracles homeobox 2 antisence (EMX2-AS) was significantly increased during the osteoblast differentiation of mesenchymal stem cells (MSCs). Overexpression of lncRNA EMX2-AS promoted osteogenesis in vitro and enhanced heterotopic bone formation in vivo, whereas lncRNA EMX2-AS knockdown had the opposite effect. EMX2 could negatively regulate the osteoblast differentiation of MSCs. lncRNA EMX2-AS was 80% expressed in the cytoplasm during osteoblast differentiation in MSCs. Mechanistic analysis revealed that lncRNA EMX2-AS acts as a positive regulator of osteogenic differentiation through interaction with EMX2 and suppression of its expression at the translational level and Wnt/*β*-catenin pathway is involved in lncRNA EMX2-AS/EMX2 regulated osteogenic differentiation. Our findings not only provide new targets for the treatment of diseases related to osteoblast differentiation disruption but also enrich the understanding of the regulation mechanisms of lncRNA during stem cell differentiation.

## 1. Introduction

Bone formation or osteogenesis is a very important process for the treatment of osteoblast differentiation-related disorders, including osteoporosis, artery calcification, femoral head necrosis, and bone hyperplasia. It is a multiple-step and multiple-factor-involved process, including matrix secretion and calcium mineralization by osteoblasts derived from mesenchymal stem cells (MSCs) and absorption of calcium and phosphorus by osteoclasts derived from hematopoietic stem cells [1]. MSCs have the potential for multispectral differentiation and are extremely useful cell source of medical therapy to regenerate or repair a wide variety of mature tissues, such as bone tissue [2].

Given the propensity of MSCs to undergo stochastic specification, a great deal of investigation has focused on controlling MSCs differentiation into specific lineages or regulate lineage commitment by modulating cell signaling pathways or lineage-specific transcription factors. Greater understanding of the transcriptional factors and the regulators involved in the commitment of MSCs to defined lineages has led to novel discoveries, such as the fact that these cells can transdifferentiate or switch phenotypes, from adipocyte to osteoblast [3]. The relative differentiation plasticity of these mesenchymal lineage cells is achieved by the regulation of several levels of phenotypic transcription factors. The process of osteoblast differentiation of MSCs is mainly regulated by tissue-specific transcription factors and epigenetic regulators [4, 5]. On one hand, incorporation of the induction of growth factors and cell signaling pathway–related regulators, such as bone morphogenetic proteins (BMPs)/Smads [6], mitogen-activated protein kinase (MAPK)/p38 [7], and Wnt/*β*-catenin [8], and tissue-specific transcription factors runt-related transcription factor 2 (RUNX2) [9] and Osterix [10], are activated, increasing the expression of osteoblast-specific genes alkaline phosphatase (ALP), osteocalcin (OCN), osteopontin (OPN), and collagen, type I, alpha 1 (COL1A1); eventually, MSCs differentiate into osteoblasts. On the other hand, epigenetic regulation also exerts an important role in the regulation of osteogenic differentiation of MSCs. DNA methylation and histone acetylation and methylation are important epigenetic mechanisms of osteogenesis [11–14]. New evidence suggests that noncoding RNAs (ncRNAs) were also important regulators of the bone formation process [15, 16].

ncRNAs, not including ribosomal and transfer RNAs (which have well-established roles in transcription), were formerly defined as “transcriptional noise”, which are a byproduct of RNA polymerase II transcription without biological function. ncRNAs comprise the majority of transcriptional output in terms of complexity; 75% of the human genome is transcribed, but only 2% encodes functional proteins [17]. The two major classes of ncRNA are defined according to their length; small ncRNAs are under 200 nt, and species 200 nt and longer are classified as long noncoding RNAs (lncRNAs). Over the last few years, the focus in the field has turned to how miRNAs may play a role in the lineage commitment and differentiation of stem cell populations. Many miRNAs, including miR-637, miR-214, miR-17-5p, miR-106a, miR-194, miR-216a, miR-26a, miR-450b, miR-185-5 p, miR-223, and miR-940 have been identified as positive or negative regulators in MSCs osteogenic differentiation and bone formation [18–27]. The roles and related mechanisms of miRNAs in bone development and balance have been well characterized and reviewed [28, 29] However, as another important noncoding RNA, the roles and functions of lncRNAs are still poorly characterized in osteoblast differentiation of MSCs.

LncRNA is the most recently identified class of ncRNA yet is the most diverse. Generally, lncRNAs are expressed at lower levels than protein-coding transcripts, and their expression is more restricted in terms of tissue specificity. Most lncRNAs are less conserved among species. It is evident from the relatively small amount of evidence generated that lncRNAs have a significant and crucial role in the maintenance of stem cell pluripotency [30], regulation of apoptosis [31], lineage commitment, and differentiation [32–35]. They are also involved in numerous diseases associated with aberrant cellular control including autoimmune, neurological, cardiovascular conditions, and cancer [36]. With emerging evidence of lncRNA involvement in normal and abnormal cell differentiation, examination of the literature characterizing the role of lncRNAs in lineage commitment of MSCs is of interest and importance. However, their roles and functions in osteoblast differentiation of MSCs remain largely unclear.

In this study, we compared expression patterns of lncRNAs before and after the osteoblast differentiation of MSCs. We found that the expression of an lncRNA with probe number p1757 was strongly increased during osteogenesis, which was named empty spiracles homeobox 2 (EMX2)-antisence (AS) in the UCSC database according to its position with neighboring genes. We demonstrated that lncRNA EMX2-AS plays an important role in osteoblast differentiation by gain- and loss-of-function experiments. Importantly, mechanistic analysis revealed that lncRNA EMX2-AS was a positive regulator of osteogenic differentiation through suppressing EMX2 translation and subsequent activation of the Wnt/*β*-catenin pathway. Understanding the role and function of this lncRNA may offer future therapeutic targets to combat osteoporosis or other osteogenic differentiation-related disorders.

## 2. Materials and Methods

### 2.1. Isolation, Culture, and Differentiation of MSCs

MSCs was a gift from the laboratory of Center of Excellence in Tissue Engineering, Institute of Basic Medical Sciences Chinese Academy of Medical Sciences, School of Basic Medicine Peking Union Medical College, which were isolated and cultured as previous articles have described [22]. All cells were used at passage 3–4.

### 2.2. Adipogenic Differentiation Potential Assay of MSCs

Adipogenesis induction medium (AM) was consisted of high-glucose Dulbecco's modified Eagle medium (DMEM) containing 10% fetal bovine serum (FBS; Gibco, USA), 0.5 mM 3-isobutyl-1-methylxanthine, 1 μM dexamethasone, and 0.1 mM ascorbic acid (Sigma–Aldrich, USA). When MSCs were 90% confluent, they were replaced with AM, after which the medium was changed every 3 days for a total of 12–15 days. Cells were fixed with 4% paraformaldehyde for 5 min and stained with filtered Oil red O solution for 30 min. Then the unbound dye was washed with water and visualized by light microscopy.

### 2.3. Osteogenic Differentiation Potential Assay of MSCs

Osteogenesis induction medium (OM) was consisted of high-glucose DMEM containing 10% FBS (Gibco, USA), 10 mM *β*-glycerophosphate, 10 nM dexamethasone, and 0.05 mM ascorbic acid (Sigma–Aaldrich, USA). When MSCs were 70% confluent, they were replaced with OM, after which the medium was changed every 3 days for a total of 12 days. The ALP staining was performed on day 6 according to the manufacturer's protocol of ALP staining kit (Institute of Hematology and Blood Diseases Hospital, Chinese Academy of Medical Sciences Tianjin, China). And the staining with Alizarin Red staining was performed on day 12.

### 2.4. Chondrogenic Differentiation Potential Assay of MSCs

Chondrogenesis induction medium (CM) was consisted of high-glucose DMEM containing 2% FBS (Gibco, USA), 10 ng/ml TGF-*β*1 (Sigma–Aldrich, USA), 1% Insulin Transferrin Selenium-A (ITS; Invitrogen), and 0.05 mM ascorbic acid (Sigma–Aldrich, USA). The trypsinized MSCs were resuspended in medium at a density of 1 × 10^7^ cells/mL, and 10 μL of the resuspended cells were dotted in each well of 24-well plates. Cells were cultured in CM for 21 days and the medium was changed every 3 days. For the Alcian Blue staining, the cell pellets were washed twice with PBS, fixed with 4% paraformaldehyde for 30 min, and then washed with water and stained with Alcian Blue solution in dark for 30 min at 37°C.

### 2.5. Flow Cytometry Assays

Cells were digested and resuspended in 50 μL of PBS and added 100 μL fixation/permeabilization solution for 20 min at 4°C. Then, cells were washed twice with PBS and incubated with the FITC-conjugated antibodies (fetal liver kinase-1 (FLK-1), CD44, CD29, CD105, CD31, CD34, CD106, and human leukoyte antigen DR (HLA-DR); BD Bioscience, USA) at 4°C for 1 h. After washing, cells were fixed with 4% paraformaldehyde. The fluorescence intensity was analyzed on Accuri C6 flow cytometers with CFlow software (Accuri Cytometers, Ann Arbor, MI).

### 2.6. RNA Isolation and Quantitative Real-Time Polymerase Chain Reaction (qRT-PCR)

Total RNA was extracted with TRIzol reagent (Invitrogen, USA) and chloroform. cDNA reverse transcription kit (Applied Biosystems, USA) was used to synthesize the first-strand cDNA. qRT-PCR was performed in triplicate using SYBR Green master mixture (Takara, Japan) and data were detected in ABI Prism 7300 System. The data were evaluated by 2^−*ΔΔCt*^ method and normalized to *β*-actin.

### 2.7. Western Blot Analysis

Cells were harvested with RIPA lysis buffer (Beyotime, China) with 1 mM PMSF. The supernatant concentration was detected by using the BCA Protein Assay Kit (Beyotime, China) and denatured in boiling water. Protein was separated by 10% SDS–PAGE and transferred to polyvinylidene difluoride membranes (0.22 μm, Millipore, USA). The membrane was blocked and then incubated with primary antibodies at 4°C overnight. Then, the membrane was incubated with horseradish peroxidase (HRP)-coupled second antibodies for 1 h at room temperature. Signals were visualized with an Immobilon Western Chemiluminescent HRP Substrate (Millipore, GER) and visualized on Tanon-5200 Chemiluminescent Imaging System (Tanon Science & Technology, China). The antibodies were as follows: ALP polyclonal antibody (Abcam, #ab108337), OPN polyclonal antibody (Abcam, #ab69498), RUNX2 polyclonal antibody (Cell Signaling Technology, #8486), IBSP polyclonal antibody (Cell Signaling Technology, #5468), BMPR2 polyclonal antibody (Cell Signaling Technology, #6979), EMX2 polyclonal antibody (Santa Cruz Biotechnology, # SC-28221), *β*-catenin polyclonal antibody (Abcam, # ab32572), and GAPDH polyclonal antibody (proteintech, # 60004-1-Ig).

### 2.8. Short Interfering RNA (siRNA) Transfection and Lentiviral Vector Infection

Synthetic siRNAs were purchased from Gene Biotechnology and were transfected into cells with Lipofectamine 3000 (Invitrogen, USA). Cells were harvested and the interference efficiency of cells was detected after 48 h. The full-length lncRNA EMX2-AS (GenBank accession number NR_002196.1) cDNA was cloned into the LV5-EF1-a-EGFP-Puro vector plasmid and produced according to the protocol from GenePharma (www.genepharma.com). After infection with lentivirus (MOI = 10) and selection with puromycin, almost all the cells were GFP-positive.

### 2.9. In Vivo Heterotopic Bone Formation Assay

Approximately 2 × 10^6^ cells infected with lenti-EMX2-AS or lenti-NC were loaded in HA/TCP scaffolds (porosity: 60% ± 10%, aperture: 200–500 μm, and *φ*: 2–4 mm × 5 mm; National Engineering Research Center for Biomaterials, China), incubated at 37°C overnight, and then implanted into NOD/SCID mice subcutaneously. After 12 weeks, xenografts were harvested, fixed in 4% paraformaldehyde for 1 day, and then decalcified in 10% EDTA for approximately 12 days. The osteoid formation was observed using Hematoxylin eosin staining (HE), Safranin O-fast green staining (Safranin O-fast green), and Masson's trichrome staining (Masson). The staining steps were performed according to the protocol from *SERVICEBIO* (http://www.servicebio.cn/).

### 2.10. Nuclear/Cytoplasmic Fractionation

The experiment was performed according to the NE-PER Nuclear and Cytoplasmic Extraction Reagent kit (Thermo scientific, USA), all steps and reagents were performed at low temperature. First, cells were collected and washed with PBS. CER I was added to the cellular components, vortexed and incubated on ice to extract the cytoplasm components fully. Then, added CER II and after a short centrifugation, the supernatant was collected as the cytoplasmic fraction and the pellet was considered the nuclear extract.

### 2.11. Rapid-Amplification of cDNA Ends (RACE)

RNA ligase mediated (RLM)-RACE was performed using the FirstChoice RLM-RACE kit (Ambion) to determine the 5′ and 3′ ends of lncRNA EMX2-AS. For the 5′ RACE, total RNA from MSCs was treated with calf intestine alkaline phosphatase (CIP) to remove free 5′-phosphates from noncapped transcripts and then treated with tobacco acid pyrophosphatase (TAP), leaving a 5′-monophosphate transcript exclusively from intact 5′ transcripts. A 45 bp RNA adapter was ligated to the RNA using T4 RNA ligase, then random-primed reverse transcription reaction and nested PCR amplification of the 5′ end of a specific transcript was performed. For the 3′ RACE, first strand cDNA was synthesized using the 3′ RACE adapter. The cDNA was then subjected to PCR using a lncRNA EMX2-AS specific primer and one of the 3′ RACE primers, which were complementary to the anchored adapter.

### 2.12. RNA Immunoprecipitation (RIP)

As described in the manufacturer's protocol of Magna RIP RNA-Binding Protein Immunoprecipitation Kits (Merck Millipore, Germany), specific antibodies were incubated with protein A/G beads for 30 min and 900 µL of RIP immunoprecipitation buffer (860 μL of RIP wash buffer, 35 μL of 0.5 M EDTA, and 5 μL of RNase inhibitor) was added. Cells were washed with ice-cold PBS and lysed with complete RIP lysis buffer (100 μL of RIP lysis buffer, 0.5 μL of protease inhibitor cocktail, and 0.25 μL of RNase inhibitor) and centrifuged at 14,000 rpm for 10 min at 4°C. Then, 100 µL of the supernatant was removed and incubated with the beads-antibody complex in RIP immunoprecipitation buffer for 3 h at 4°C. The supernatant was then discarded and the beads were washed six times, followed by incubation in 150 µL of proteinase K buffer (117 μL of RIP wash buffer, 15 μL of 10% SDS, and 18 μL of proteinase K) at 55°C for 30 min to digest the protein. LncRNA EMX2-AS enrichment was examined using qRT-PCR. IgG and SNRNP70 enrichment served as negative and positive controls, respectively.

### 2.13. RNA Pull-Down Assay

The preparation of biotin-labelled RNA probes was completed in vitro. RNA was transcribed from cDNA with T7 RNA polymerase (Promega) and Biotin RNA Labeling Mix (Roche) and then treated with RNase-free DNase I (Promega). Then, 1 µg of biotin-labelled RNAs in RNA structure buffer (10 mM Tris pH 7, 10 mM MgCl_2_, and 0.1 M KCl) was heated at 95°C for 2 min, put on ice for 3 min, and then left at room temperature for 30 min to allow proper secondary structure formation. Approximately 50 pM RNA probes were incubated with 50 µL of washed streptavidin agarose beads (Invitrogen) at RT for 30 min and further incubated with 20–200 µg MSCs cytoplasmic extract (the concentration of proteins ≥2 mg/mL) at 4°C for 1 h. The beads-RNA-complex was washed briefly and then 50 µL of elution buffer was added and the sample was vortexed for 15 min. Next, the supernatant components were detected by western blot or separated by SDS–PAGE and silver staining, followed by mass spectrometry (MS) identification.

### 2.14. Statistical Analysis

Each experimental procedure involving cells was repeated at least three times. And quantitative data were presented as the mean ± standard deviation (SD) (*n* = 3). Comparison between groups were performed with one-way analysis of variance (ANOVA) test or Student's *t* test. *P* < 0.05 was considered as statistically significant, *⁣*^*∗*^*p* < 0.05, *⁣*^*∗∗*^*p* < 0.01, *⁣*^*∗∗∗*^*p* < 0.001, and *⁣*^*∗∗∗∗*^*p* < 0.0001.

## 3. Results

### 3.1. Identification and Characterization of MSCs

**Figure 1:**
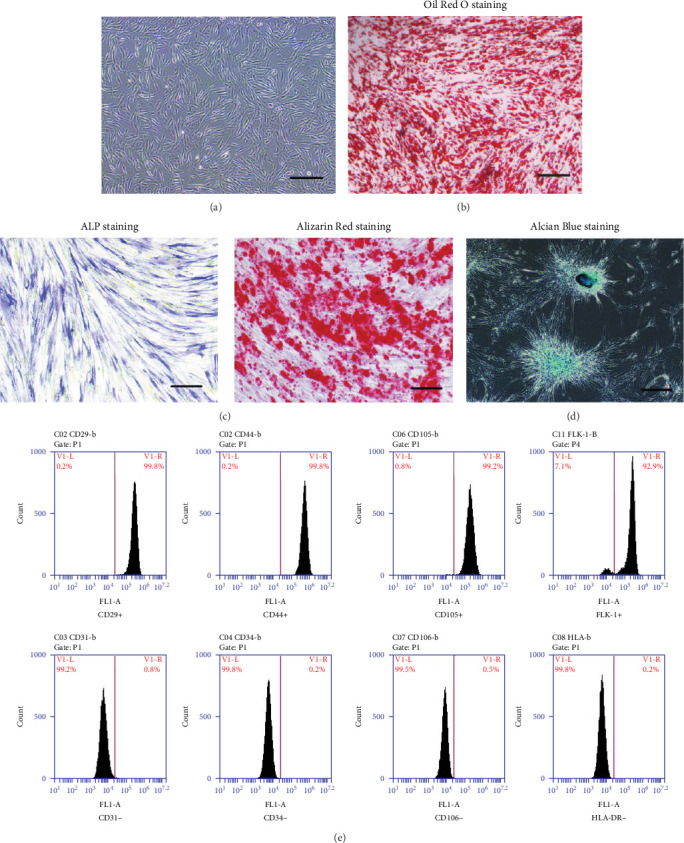
Identification and characterization of MSCs. (a) The morphology of MSCs observed under light microscope. (b) The adipogenic differentiation of MSCs was confirmed by Oil Red O staining. (c) The osteogenic differentiation of MSCs was identified by ALP staining on day 6 and Alizarin Red staining on day 12 for osteoblasts. (d) The chondrogenic differentiation of MSCs was detected by Alcian Blue staining on day 21. (e) Flow cytometry assay detected cell surface marker expression of MSCs. All the staining and data were confirmed by over three repeated trials. Scale bar: 100 μm. ALP, alkaline phosphatase; FLK-1, fetal liver kinase-1; HLA-DR, human leukoyte antigen DR; MSCs, mesenchymal stem cells.

MSCs isolated from adipose tissue were cultured and expanded in DMEM-F12 medium. After two expansion cycles, cells changed to possess a homogenously spindle-like morphology and subconfluently grew in a monolayer (Figure 1a). Under conditional medium, they exhibited adipogenesis, osteogenesis, and chondrogenesis potential, which was indicated by Oil Red O staining (Figure 1b), ALP and Alizarin Red staining (Figure 1c), and Alcian Blue staining (Figure 1d), respectively. We detected the surface markers of cells by flow cytometry assay. Cells expressed high levels of CD29, CD44, CD105, and FLK-1 and were negative for the endothelial marker CD31, the bone marrow MSCs maker CD106, and the hematopoietic markers CD34 and HLA-DR (Figure 1e). These results illustrate that we successfully isolated MSCs from adipose tissue.

### 3.2. lncRNA EMX2-AS is Upregulated During Osteoblast Differentiation of MSCs

**Figure 2:**
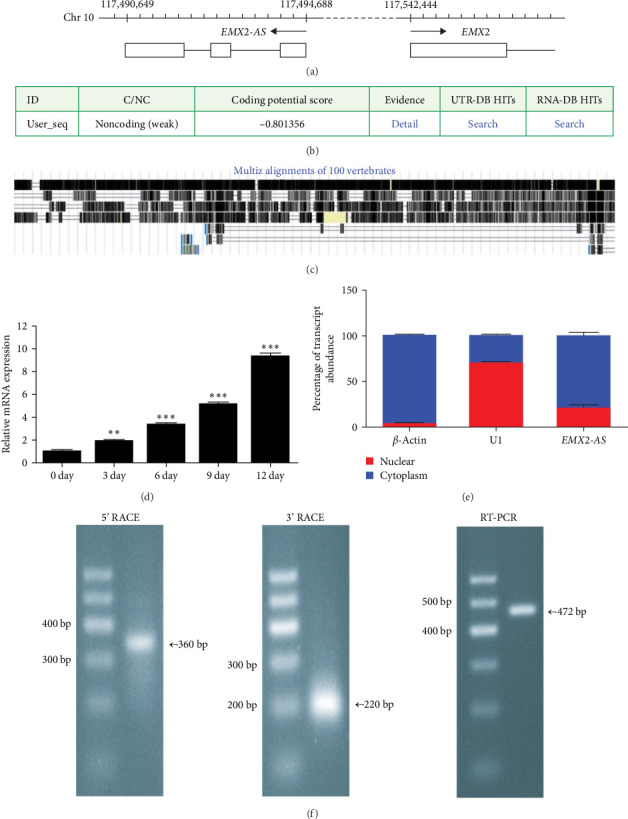
The identification and dynamic expression pattern of lncRNA EMX2-AS during osteoblast differentiation of MSCs. (a) The location of EMX2-AS and EMX2 in the genome. (b) The protein-coding potencial of lncRNA EMX2-AS (http://cpc.cbi.pku.edu.cn). (c) Conservation analysis of lncRNA EMX2-AS (UCSC Genome Browser). (d) The dynamic expression pattern of lncRNA EMX2-AS during osteogenic differentiation of MSCs was validated by qRT-PCR. (e) Nucleocytoplasmic fractionation of MSCs was performed and followed by PCR. (f) The full-length of lncRNA EMX2-AS was detected by 5′ RACE, 3′ RACE, and PCR. *β*-actin RNA was used as a cytoplasmic location control and U1 was served as a nuclear location control. Quantitative data are presented as the mean ± SD. *n* = 3. EMX2-AS, empty spiracles homeobox 2 antisence; lncRNA, long noncoding RNA; MSCs, mesenchymal stem cells; qRT-PCR, quantitative real-time polymerase chain reaction; RACE, rapid-amplification of cDNA ends; SD, standard deviation.

Arraystar Human LncRNA Microarray V3.0 was conducted to identify lncRNAs that were involved in the osteoblast differentiation of MSCs. The expression pattern of lncRNAs was compared between MSCs and cells that had 3 days of osteogenic induction. According to *p* values and fold change, we selected some significantly upregulated lncRNAs to investigate. The change in expression of lncRNA EMX2-AS was most significant, thus, we chose lncRNA EMX2-AS to evaluate in follow-up experiments. lncRNA EMX2-AS is an antisense lncRNA of protein-coding gene EMX2 (Figure 2a). We then assessed the protein-coding abilities of lncRNA EMX2-AS at http://cpc.cbi.pku.edu.cn, the coding potenical score is −0.801356, indicating EMX2-AS is ncRNA (Figure 2b). Further interspecies conservative analysis in the UCSC Genome Browser revealed that lncRNA EMX2-AS is not conserved in humans and mouse (Figure 2c). qRT-PCR further validated that lncRNA EMX2-AS expression increased during osteogenic differentiation and exhibited up to tenfold greater expression in 9 day induced cells compared to day 0 (Figure 2d). Nucleocytoplasmic fractionation of MSCs indicated that approximately 80% lncRNA EMX2-AS was located in the cytoplasm (Figure 2e), which indicated that the regulation of lncRNA EMX2-AS may function at the post-transcriptional level. Using RACE, we obtained a 360-bp sequence at the 5′ end and a 220-bp sequence at the 3′ end and performed PCR to obtain its full sequence, which is 472-bp in length (Figure 2f).

### 3.3. Decreased Expression of lncRNA EMX2-AS Inhibits the Osteoblast Differentiation of MSCs

**Figure 3:**
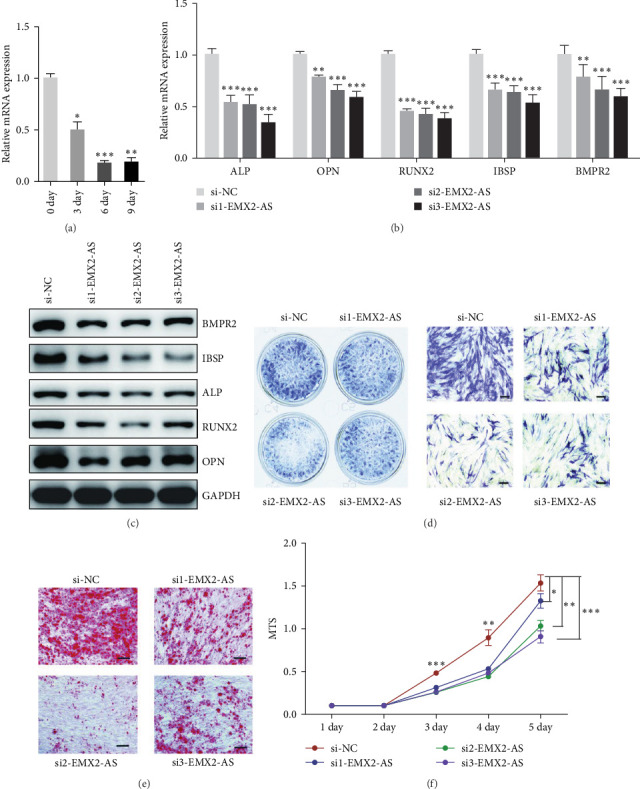
Interfering the expression of lncRNA EMX2-AS inhibits the osteogenic differentiation of MSCs. (a) qRT-PCR analysis detected the mRNA level of lncRNA EMX2-AS after siRNA transfection. (b, c) qRT-PCR and western blot analyses detected the expression level of osteogenic transcription factors and marker genes ALP, OPN, RUNX2, IBSP, and BMPR2 on day 6 of osteogenic differentiation. (d) ALP staining was performed on day 4 of osteogenic differentiation to view the efficiency of osteogenesis. (e) Calcium deposition was analyzed by Alizarin Red staining on day 12 of osteogenic differentiation. (f) Cell proliferation of MSCs was measured by MTS assay. *β*-Actin was used as an internal control in qRT-PCR and GAPDH was used as an internal control in western blot analyses. Quantitative data are presented as the mean ± SD. *n* = 3, *⁣*^*∗*^*p* < 0.05; *⁣*^*∗∗*^*p* < 0.01; *⁣*^*∗∗∗*^*p* < 0.001 compared with the control. All staining and western blot images were confirmed by over three repeated trials. Scale bar: 100 μm. ALP, alkaline phosphatase; BMP, bone morphogenetic protein; EMX2-AS, empty spiracles homeobox 2 antisence; lncRNA, long noncoding RNA; MSCs, mesenchymal stem cells; OPN, osteopontin; qRT-PCR, quantitative real-time polymerase chain reaction; RUNX2, runt-related transcription factor 2; SD, standard deviation.

The endogenous expression of lncRNA EMX2-AS was inhibited by three siRNAs and then induced in osteogenic medium. qRT-PCR analysis showed that all of the three siRNAs decreased lncRNA EMX2-AS expression significantly (Figure 3a). qRT-PCR and western blot analyses indicated that downregulated lncRNA EMX2-AS could decrease osteogenic transcription factors and marker genes RUNX2, OPN, ALP, IBSP, and BMPR2 levels (Figure 3b,c). Low expression of lncRNA EMX2-AS decreased number of ALP staining positive cells (Figure 3d). Consistently, Alizarin Red staining on day 12 of osteoblast differentiation showed that calcium deposition in lncRNA EMX2-AS downregulated cells was less than that in control cells (Figure 3e). Furthermore, we found that downregulated EMX2-AS expression delayed the cell proliferation that was closely related to osteogenic differentiation (Figure 3f). These results suggested that EMX2-AS might an important regulator in osteoblast differentiation.

### 3.4. Overexpression of lncRNA EMX2-AS Promotes Osteogenesis and Enhances Heterotopic Bone Formation

**Figure 4:**
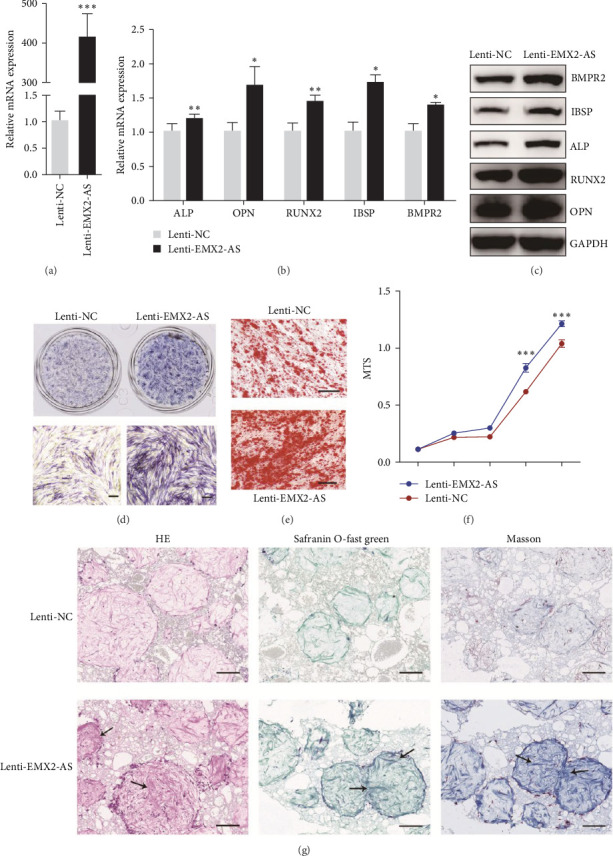
Overexpression of lncRNA EMX2-AS promotes osteogenic differentiation and heterotopic bone formation. An overexpressing lncRNA EMX2-AS precursor lentiviral vector (lenti-EMX2-AS) was used to upregulate lncRNA EMX2-AS expression and the negative control (lenti-NC) expressed. (a) Scrambled sequence and qRT-PCR analysis detected the mRNA level of lncRNA EMX2-AS after lentiviral vector infection. (b, c) qRT-PCR and western blot analyses detected the mRNA and protein levels of marker genes ALP, OPN, RUNX2, IBSP, and BMPR2 on day 6 of osteogenic differentiation. (d) ALP staining viewed the efficiency of osteogenesis on day 4 of osteogenic differentiation. (e) Calcium deposition was indicated by Alizarin Red staining on day 12 of osteogenic differentiation. (f) MTS assay was used to measure cell proliferation of MSCs. (g) The osteoid formation in xenografts of mice were detected using HE, Safranin O-fast green staining (Safranin O-fast green), and Masson's Trichrome staining (Masson). Black arrows indicated bone matrix. *β*-actin was used as an internal control in qRT-PCR and GAPDH was used as an internal control in western blot analysis. Quantitative data are presented as the mean ± SD. *n* = 3, *⁣*^*∗*^*p* < 0.05; *⁣*^*∗∗*^*p* < 0.01; *⁣*^*∗∗∗*^*p* < 0.001 compared with the control. All the staining and western blot images were confirmed by over three repeated trails. Scale bar: 100 μm. ALP, alkaline phosphatase; BMP, bone morphogenetic protein; EMX2-AS, empty spiracles homeobox 2 antisence; HE, hematoxylin eosin staining; lncRNA, long noncoding RNA; MSCs, mesenchymal stem cells; OPN, osteopontin; qRT-PCR, quantitative real-time polymerase chain reaction; RUNX2, runt-related transcription factor 2; SD, standard deviation.

To further investigate the effect of lncRNA EMX2-AS on MSCs osteogenesis, cells were infected with the lncRNA EMX2-AS precursor in a lentiviral vector (lenti-EMX2-AS) or a scrambled sequence control (lenti-NC) and were put under puromycin selection until almost all cells were GFP-positive. qRT-PCR analysis confirmed that lncRNA EMX2-AS mRNA level was significantly increased in lenti-EMX2-AS infected cells (Figure 4a). Osteogenic transcription factors and marker genes RUNX2, OPN, ALP, IBSP, and BMPR2 were significantly upregulated in lenti-EMX2-AS infected cells compared with lenti-NC infected cells, detected by qRT-PCR and western blot analyses (Figure 4b,c). The ALP staining on day 4 of osteogenic induced cells and Alizarin Red staining on day 12 of osteogenic induced cells were both enhanced (Figure 4d,e). The proliferation of MSCs was accelerated in lenti-EMX2-AS infected cells (Figure 4f).

To examine whether overexpression of lncRNA EMX2-AS could also enhance heterotopic bone formation in vivo, a classic ectopic bone formation model in NOD/SCID mice was used. MSCs infected with lenti-EMX2-AS or lenti-NC, were loaded in HA/TCP scaffolds and implanted subcutaneously into 6-week-old male NOD/SCID mice for 12 weeks. HE and Safranin O-fast green staining showed a significant increase in osteoid formation in xenografts that were incubated with lenti-EMX2-AS infected cells. Likewise, collagen I, which can be stained blue by Masson's trichrome staining (Masson), had greater expression in the xenografts that were incubated with lenti-EMX2-AS infected cells (Figure 4g). Together, these data demonstrate that overexpression of lncRNA EMX2-AS not only promotes osteogenic differentiation in vitro, but also enhances heterotopic bone formation in vivo.

### 3.5. lncRNA EMX2-AS Interacts with EMX2

**Figure 5:**
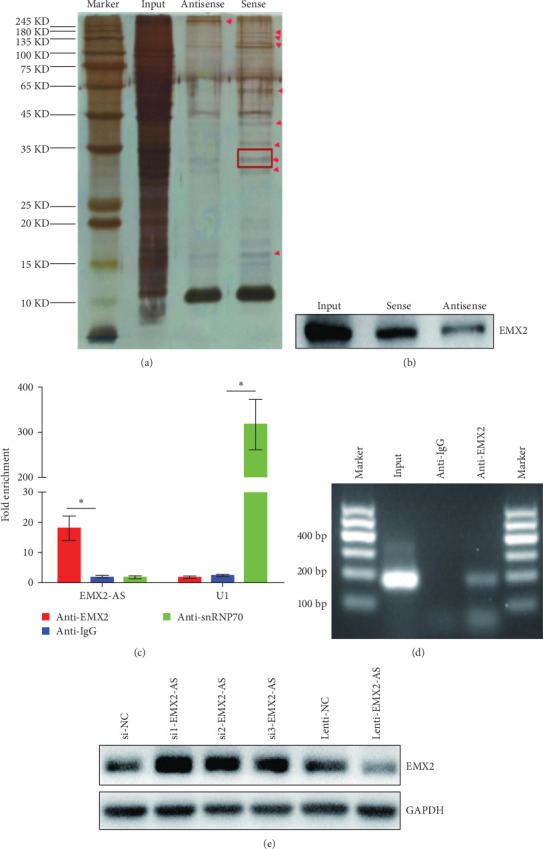
lncRNA EMX2-AS interacts with EMX2. (a) Biotinylated lncRNA EMX2-AS sense and antisense RNA probes were used in an RNA pull-down assay, followed by silver staining and MS. Red box denotes EMX2. (b) Immunoblot analyzed the EMX2 protein after RNA pull-down assay. (c) RIP assay with anti-EMX2 antibody and quantification of the retrieved RNA by qRT-PCR. Anti-IgG antibody was used as negative control and anti-snRNP70 antibody was used as the positive control. (d) Agarose gel electrophoresis analyzed the enrichment of lncRNA EMX2-AS in amplification products. (e) Western blot analysis detected the EMX2 protein after lncRNA EMX2-AS was downregulated or overexpressed. Quantitative data are presented as the mean ± SD. *n* = 3, *⁣*^*∗*^*p* < 0.05 compared with the control. EMX2-AS, empty spiracles homeobox 2 antisence; lncRNA, long noncoding RNA; MS, mass spectrometry; qRT-PCR, quantitative real-time polymerase chain reaction; RIP, RNA immunoprecipitation; SD, standard deviation.

As lncRNA EMX2-AS was mainly located in the cytoplasm, we suspected that lncRNA EMX2-AS may function at the posttranscriptional level. To confirm this, we performed an RNA pull-down assay. Biotinylated sense and antisense RNA probes were transcribed in vitro and incubated with streptavidin beads and cytoplasm extract. The precipitated proteins were evaluated by MS and western blot. Silver staining showed one differential band approximately 30 kDa appeared in the sense probe precipitated sample (Figure 5a). MS indicated it was the EMX2 protein, which was further confirmed by western blot analysis (Figure 5b). Next, we performed an RIP assay with the EMX2 antibody and then assayed the enrichment by quantitative PCR using specific primers for the lncRNA EMX2-AS. The lncRNA EMX2-AS transcript was enriched in the anti-EMX2 antibody sample but not in the anti-IgG antibody control. U1 was pulled down by the snRNP70 antibody, which was used as a positive control (Figure 5c). The enrichment of lncRNA EMX2-AS in amplification products was also analyzed by agarose gel electrophoresis (Figure 5d). We also found that the level of EMX2 protein was increased in lncRNA EMX2-AS siRNA transfected cells and decreased in lncRNA EMX2-AS overexpressed cells (Figure 5e). These data further confirmed that lncRNA EMX2-AS could interact with EMX2 in MSCs cells and regulate EMX2 protein level.

### 3.6. Knockdown of EMX2 Mimics the Promoting Effects of lncRNA EMX2-AS on Osteogenesis

**Figure 6:**
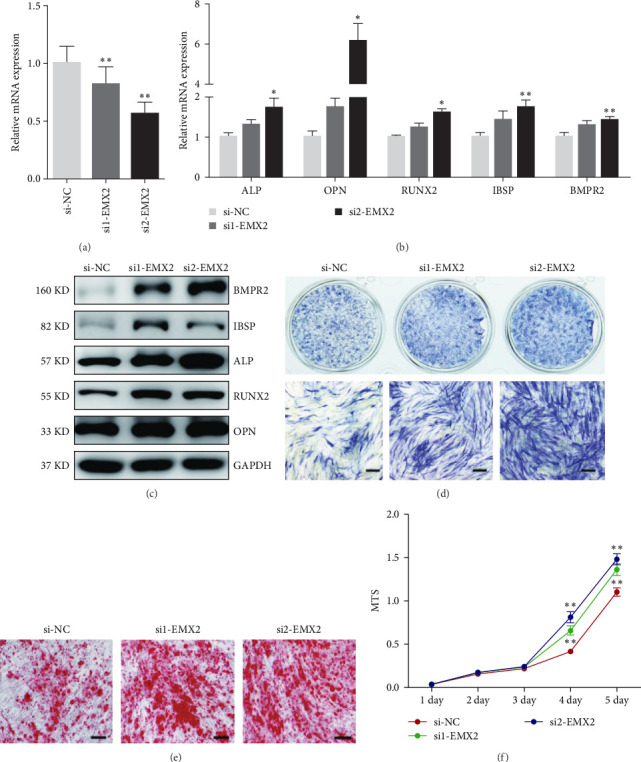
Knockdown of endogenous EMX2 increases osteogenic differentiation of MSCs. (a) qRT-PCR analysis detected the mRNA level of EMX2 after siRNA transfection. (b, c) qRT-PCR and western blot analyses detection of the expression of osteogenic transcription factors and markers ALP, OPN, RUNX2, IBSP, and BMPR2 on day 6 of osteogenic differentiation. (d) ALP staining was performed on day 4 of osteogenic differentiation to view the efficiency of osteogenesis. (e) Calcium deposition was indicated by Alizarin Red staining on day 12 of osteogenic differentiation. (f) MTS assay was used to measure cell proliferation of MSCs. *β*-actin was used as an internal control in qRT-PCR and GAPDH was used as an internal control in western blot analysis. Quantitative data are presented as the mean ± SD. *n* = 3, *⁣*^*∗*^*p* < 0.05; *⁣*^*∗∗*^*p* < 0.01; *⁣*^*∗∗∗*^*p* < 0.001 compared with the control. All staining and western blot images were confirmed by over three repeated trails. Scale bar: 100 μm. ALP, alkaline phosphatase; BMP, bone morphogenetic protein; MSCs, mesenchymal stem cells; OPN, osteopontin; qRT-PCR, quantitative real-time polymerase chain reaction; RUNX2, runt-related transcription factor 2; SD, standard deviation.

So far, it is unclear whether EMX2 regulates osteogenic differentiation. To examine whether EMX2 could regulate the osteogenic differentiation of MSCs, we chose two siRNAs to inhibit the endogenous EMX2 expression. qRT-PCR analysis confirmed that both of them decreased EMX2 expression compared with the negative control (Figure 6a). Using qRT-PCR and western blot analysis, we found that the osteogenic transcription factors and marker genes RUNX2, OPN, ALP, IBSP, and BMPR2 were significantly upregulated in EMX2 siRNAs transfected cells (Figure 6b,c). The ALP staining positive cells on day 4 of osteogenic differentiation and the Alizarin Red staining positive cells on day 12 were both increased (Figure 6d,e). In addition, the proliferation of MSCs was increased in EMX2 siRNA transfected cells compared with the negative control (Figure 6f). These data hinted that EMX2 could negatively regulate the osteogenic differentiation of MSCs and that the knockdown of endogenous EMX2 could mimic the promoting effect of lncRNA EMX2-AS on the osteogenic differentiation of MSCs, which further confirmed that lncRNA EMX2-AS may interact with EMX2 to regulate osteogenesis in MSCs.

### 3.7. Wnt/*β*-Catenin Pathway Is Involved in lncRNA EMX2-AS/EMX2 Regulated Osteogenic Differentiation

**Figure 7:**
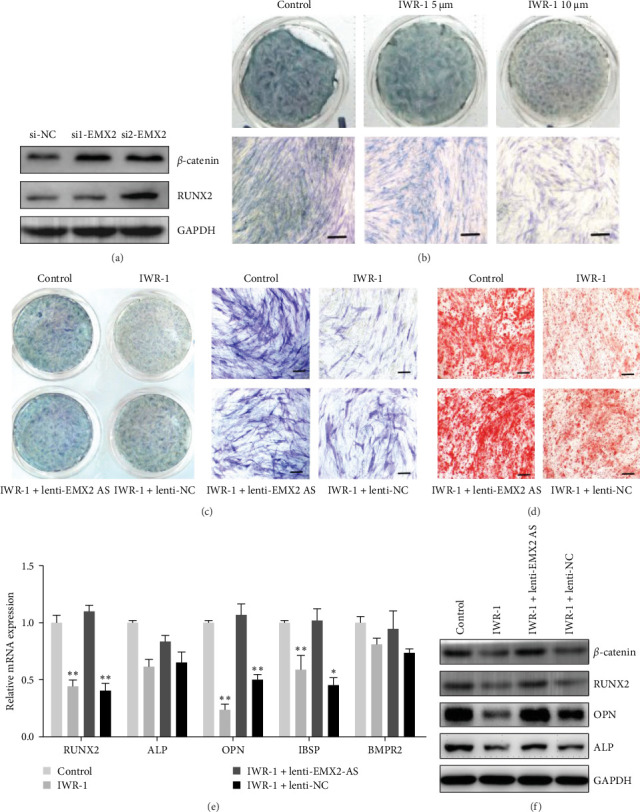
Overexpression of lncRNA EMX2-AS mitigated the inhibition of EMX2 on the Wnt/*β*-catenin pathway. (a) Western blot analyses detected the expression level of *β*-catenin and RUNX2. (b) ALP staining was performed on day 4 of osteogenic differentiation to view inhibitory effect of different concentrations of WNR-1 on osteogenesis. (c, d) ALP staining showed the osteogenesis of MSCs and Alizarin Red staining. Alizarin Red staining showed the calcification. (e) qRT-PCR analysis detected the mRNA level of marker genes ALP, OPN, RUNX2, IBSP, and BMPR2 on day 6 of osteogenic differentiation. (f) Western blot analyses detected the expression level of *β*-catenin and transcription factors RUNX2 and marker genes ALP, OPN, and *β*-actin was used as an internal control in qRT-PCR and GAPDH was used as an internal control in western blot analyses. IWR-1:Wnt/*β*-catenin inhibitor (HY-12238, MedChemExpress). Quantitative data are presented as the mean ± SD. *n* = 3, *⁣*^*∗*^*p* < 0.05; *⁣*^*∗∗*^*p* < 0.01; *⁣*^*∗∗∗*^*p* < 0.001 compared with the control. All staining and western blot images were confirmed by over three repeated trials. Scale bar: 100 μm. ALP, alkaline phosphatase; BMP, bone morphogenetic protein; EMX2-AS, empty spiracles homeobox 2 antisence; lncRNA, long noncoding RNA; MSCs, mesenchymal stem cells; OPN, osteopontin; qRT-PCR, quantitative real-time polymerase chain reaction; RUNX2, runt-related transcription factor 2; SD, standard deviation.

Next, we examined the pathway involved in lncRNA EMX2-AS/EMX2-regulated osteogenic differentiation. According to website prediction and western bolt results, the expression of *β*-catenin was upregulated after EMX2 decreased (Figure 7a).To evaluate the effects of the Wnt/*β*-catenin pathway on lncRNA EMX2-AS regulated osteogenic differentiation, we used different concentrations of IWR-1 (an inhibitor of Wnt pathway) treated MSC 24 h and induce osteogenic differentiation. ALP staining showed that 5 and 10 μM IWR-1 both reduced osteogenic differentiation (Figure 7b). Then MSCs infected with lenti-EMX2-AS or with control vector lenti-NC were treated with IWR-1 (5 μM), and the osteogenesis efficiency was analyzed. The ALP stainning and Alizarin Red staining showed the osteogenic inhibition induced by IWR-1 can be reversed by overexpression of lncRNA EMX2-AS (Figure 7c,d). Western blot results showed that the decrease of *β*-catenin induced by IWR-1 could also be achieved by increasing lncRNA EMX2-AS expression, so as the osteogenic transcription factors RUNX2 and marker genes OPN and ALP (Figure 7e). qRT-PCR analyses also showed the expression of osteogenic marker was reversed in the lenti-EMX2-AS + IWR-1 group (Figure 7f). Above data suggested that lncRNA EMX2-AS promotes MSCs osteogenic differentiation possibly by the Wnt/*β*-catenin pathway.

## 4. Discussion

Understanding the mechanism of lncRNA action in lineage commitment and differentiation is important not only for normal tissue and organ development but also for disease states. However, the mechanisms of lncRNA action in osteoblast differentiation and bone formation of MSCs remain largely unknown. Here, we found that lncRNA EMX2-AS plays an important role in the osteoblast differentiation process of MSCs. lncRNA EMX2-AS acts as a positive regulator of osteoblast differentiation through suppressing EMX2 protein expression at the translational level.

lncRNAs have gained widespread attention in recent years as a potentially new and crucial layer of biological regulation. They have a significant and crucial role in the maintenance, commitment and differentiation of MSCs to mature lineages. Numerous lncRNAs are known to be involved in carcinogenesis at the transcriptional or posttranscriptional levels and some lncRNAs have been reported to regulate the osteogenic differentiation of stem cells [37, 38]. MEG3 has been demonstrated to upregulate miR-133a-3p and inhibit osteogenic differentiation in bone marrow MSCs from patients with postmenopausal osteoporosis, which also plays an essential role in osteogenic differentiation in bone marrow MSCs, partially by activating BMP4 transcription [39, 40]. H19, acting as an endogenous competitive ceRNA for miR-141 and miR-22, thereby, promoting osteogenic differentiation [41]. H19 is also the precursor of miR-675, which not only downregulates TGF-*β*1 but also inhibits Smad3 phosphorylation and downregulates HDAC4/5 leading to a reduction of HDACs recruitment to the promoter of osteogenic specific RUNX2 [42]. MALAT1 has been shown to positively regulate the expression of Smad4 through sponging miR-204 and promotes osteogenic differentiation of primary valve interstitial cells (VICs) [43]. TUG1 positively regulates the expression of RUNX2 through sponging miR-204-5p and promotes osteogenic differentiation [44]. Recently, more studies reports lncRNAs are important moderators in osteogenesis. lnc-PPP2R1B mediated alternative splicing of PPP2R1B through retaining exon 2 and 3 by interacting with heterogeneous nuclear ribonucleoprotein L like (HNRNPLL) and then promoted osteogenesis [45]. lncRNA RAD51-AS1 is low expressed in hBMSCs from osteoporotic patients and found that RAD51-AS1 promoted the proliferation and osteogenic differentiation of BMSCs by binding YBX1, inhibiting the translation of Smad7 and Smurf2, and transcriptionally upregulated PCNA and SIVA1 [46].

To explore some lncRNAs that play an important role in osteoblast differentiation and bone formation of MSCs, we compared the lncRNA expression profiles of undifferentiated and differentiated cells during osteogenesis of MSCs through high-throughput Arraystar Human LncRNA Microarray. We selected lncRNAs that were significantly upregulated after differentiation of MSCs into osteoblasts, which could play an important role in the osteogenic differentiation process. We demonstrated that the knockdown of lncRNA with the probe number p1757 significantly inhibited the differentiation of MSCs into osteogenic lineage. By retrieving the annotation of the lncRNA on the UCSC, we found that the lncRNA corresponding to p1757 was named EMX2OS. It is an antisense of EMX2, also named EMX2-AS, and the length is 472 bp. As early as 2003, EMX2OS was identified in the genome and was reported as another polyadenylated transcript that is transcribed on the strand opposite to EMX2 and overlaps with the EMX2 transcript [47]. Conservation of functional human and murine EMX2 antisense genes, overlap between the sense and the antisense transcripts, and identical cellular expression patterns suggest a biological function for EMX2OS, presumably in the regulation of EMX2. However, so far, there have been no reports of EMX2-AS directly regulating EMX2 expression nor has there been a clear report on EMX2-AS functions. We found that for the first time, downregulation of lncRNA EMX2-AS inhibits the osteogenic differentiation of MSCs, while overexpression of lncRNA EMX2-AS promotes osteogenesis in vitro and enhances heterotopic bone formation in vivo.

EMX2 is one of two vertebrate homologs of the *Drosophila melanogaster* empty spiracles homeodomain transcription factor [48]. Expressed in the central nervous system, EMX2 plays a critical role in brain and skeletal development [49]. EMX2 is also expressed in urogenital tissues during development, with mRNA evident in the earliest stages of differentiation in the primordia that will give rise to the kidneys, gonads, and genital tract [50]. Point mutations in human EMX2 result in severe schizencephaly [51, 52]. Pellegrini et al. have found that the scapula and ilium do not develop in Emx2 knock-out mice [53]. However, the role and the mechanism of EMX2 in the regulation of osteoblast differentiation and bone formation remain unclear now. To determine whether EMX2 has a regulatory effect on osteoblast differentiation, we used siRNA to decrease the expression of EMX2 and found that EMX2 could negatively regulate the osteoblast differentiation of MSCs, and knocking down endogenous EMX2 could mimic the promoting effect of lncRNA EMX2-AS on osteogenic differentiation, which suggests that lncRNA EMX2-AS may interact with EMX2 to regulate osteogenesis of MSCs.

Unlike miRNAs, lncRNAs can either positively or negatively regulate the expression of protein-coding genes through a variety of mechanisms. Generally, lncRNAs act as decoys, guides, and scaffolds that interfere with or enhance the function of other regulators, including proteins and miRNAs, by directly interacting with DNA, RNA, or proteins [54]. Although lncRNA EMX2-AS was annotated as an antisense nucleic acid of EMX2 because of its position with neighboring genes, so far, there has been no accurate report of lncRNA EMX2-AS directly regulating EMX2 expression. Accumulating evidence has revealed that lncRNAs can regulate gene expression either in cis or in trans in the nucleus and act as competitors or reservoirs of miRNA/mRNA to indirectly regulate gene expression in the cytoplasm [55, 56]. We analyzed the nucleocytoplasmic fractionation of MSCs cells and found that approximately 80% of lncRNA EMX2-AS was located in the cytoplasm. The currently reported ways in which cytoplasmic lncRNA regulates its target molecules include targeting mRNA for degradation or protecting it, transcription factor trafficking, translation disruption or ribosome targeting, or acting as decoy miRNA [57]. We analyzed the effect of the lncRNA EMX2-AS expression changes on EMX2 expression and found that the protein expression of EMX2 increased significantly after lncRNA EMX2-AS knockdown, but the mRNA expression did not change significantly. Our results suggest that lncRNA EMX2-AS may play a role in regulating the translation of the EMX2 protein.

To further clarify whether lncRNA EMX2-AS regulates osteoblast differentiation by directly affecting the expression of EMX2 and the possible mechanism, we analyzed the combination of EMX2 and lncRNA EMX2-AS through the RNA pull-down and RIP assays. We evaluated precipitated proteins from RNA pull-down assay by MS and western blot and found that EMX2 protein was pulled down by a biotinylated sense RNA probe of lncRNA EMX2-AS. Furthermore, we also found that the lncRNA EMX2-AS transcript was enriched in anti-EMX2 antibody sample, which was performed by RIP assay. These results demonstrate that lncRNA EMX2-AS can regulate the expression of EMX2 protein at a translational level. Previous studies showed that EMX2 expression was found to negatively regulate the Wnt pathway and loss of Emx2 function leads to ectopic expression of Wnt1 in the developing telencephalon and cortical dysplasia [58, 59]. Wnt pathway is closely related to osteogenic differentiation, and RUNX2, the key gene for osteogenic transcription was a downstream target gene of the Wnt/*β*-catenin.Our study also found an increase in *β*-catenin and RUNX2 expression after inhibition of EMX2. And Wnt inhibitor IWR-1 slowed down the process of osteogenic differentiation, which is consistent with the reported studies [60, 61]. In contrast, overexpression of lncRNA EMX2-AS could partially reverse the effects of Wnt inhibitors on *β*-catenin/RUNX2 expression and the osteogenesis efficiency. We hypothesized that lncRNA EMX2-AS disrupted *β*-catenin expression by inhibiting EMX2 protein levels, thereby mediating the regulation of osteogenic genes such as RUNX2, thus, promotes MSCs osteogenic differentiation, but the deeper mechanisms remain more research.

Summarily, in this study, we identify lncRNA EMX2-AS as a novel positive regulator of MSCs osteoblast differentiation and bone formation. Moreover, our findings demonstrate the interaction between lncRNA EMX2-AS and EMX2 in MSCs and reveal that lncRNA EMX2-AS negatively regulates the expression of EMX2 at a translational level during the osteoblast differentiation of MSCs. EMX2 regulatedWnt/*β*-catenin pathway and mediated the regulation of osteogenic genes. Our findings not only provide new targets for the treatment of diseases related to osteoblast differentiation disruption but also enrich the understanding of the regulation mechanism of lncRNA for stem cell differentiation.

## Data Availability

The datasets used or analyzed during the current study are available from the corresponding author on reasonable request.

## Nomenclature

LncRNAs: long noncoding RNAs
MSCs: mesenchymal stem cells
EMX2: empty spiracles homeobox 2
EMX2-AS: empty spiracles homeobox 2 antisence
BMPs: bone morphogenetic protein
MAPK: mitogen-activated protein kinase
Runx2: runt-related transcription factor 2
OPN: osteopontin
ALP: alkaline phosphatase
OCN: osteocalcin
COL1A1: collagen, type I, alpha 1
FLK-1: fetal liver kinase-1
HLA-DR: human leukoyte antigen DR
FBS: fetal bovine serum
RACE: rapid-amplification of cDNA ends
RIP: RNA immunoprecipitation
SD: standard deviation
ANOVA: analysis of variance
PCR: polymerase chain reaction
qRT-PCR: quantitative real-time polymerase chain reaction.
